# Migraine classification by machine learning with functional near-infrared spectroscopy during the mental arithmetic task

**DOI:** 10.1038/s41598-022-17619-9

**Published:** 2022-08-26

**Authors:** Wei-Ta Chen, Cing-Yan Hsieh, Yao-Hong Liu, Pou-Leng Cheong, Yi-Min Wang, Chia-Wei Sun

**Affiliations:** 1 Department of Neurology, Keelung Hospital,Ministry of Health and Welfare, No. 268, Xin 2nd Rd., Xinyi Dist, Keelung, 20148 Taiwan, ROC; 2 Neurological Institute, Taipei Veterans General Hospital, No. 201, Sec.2, Shipai Rd., Beitou Dist, Taipei, 112201 Taiwan, ROC; 3 Department of Photonics, College of Electrical and Computer Engineering, National Yang Ming Chiao Tung University, No. 1001, University Road, East District, Hsinchu, 300093 Taiwan, ROC; 4 Department of Pediatrics, National Taiwan University Hospital, Hisnchu Branch, No. 25, Ln. 442, Sec.1, Jingguo Rd., North Dist, Hsinchu, 30059 Taiwan, ROC; 5 Institute of Biomedical Engineering, College of Electrical and Computer Engineering, National Yang Ming Chiao Tung University, No. 1001, University Road, East District, Hsinchu, 300093 Taiwan, ROC; 6 Medical Device Innovation and Translation Center, National Yang Ming Chiao Tung University, No. 155, Sec. 2, Linong St., Beitou Dist., Taipei, 112304 Taiwan, ROC; 7 Department of Biological Science and Technology, National Yang Ming Chiao Tung University, Hsinchu, Taiwan

**Keywords:** Diseases of the nervous system, Diagnostic markers, Biomedical engineering

## Abstract

Migraine is a common and complex neurovascular disorder. Clinically, the diagnosis of migraine mainly relies on scales, but the degree of pain is too subjective to be a reliable indicator. It is even more difficult to diagnose the medication-overuse headache, which can only be evaluated by whether the symptom is improved after the medication adjustment. Therefore, an objective migraine classification system to assist doctors in making a more accurate diagnosis is needed. In this research, 13 healthy subjects (HC), 9 chronic migraine subjects (CM), and 12 medication-overuse headache subjects (MOH) were measured by functional near-infrared spectroscopy (fNIRS) to observe the change of the hemoglobin in the prefrontal cortex (PFC) during the mental arithmetic task (MAT). Our model shows the sensitivity and specificity of CM are 100% and 75%, and that of MOH is 75% and 100%.The results of the classification of the three groups prove that fNIRS combines with machine learning is feasible for the migraine classification.

## Introduction

Different from general headaches, migraine attacks are usually accompanied by continuous pulsations which last for 4 to 72 h^1^. About 60% of the patients have premonitory symptoms 2 to 72 h before the attack of migraine, such as drowsiness, tiredness, sensitivity to light, noise and certain odors; 40% of the patients have aura before the attack of migraine, such as blurred vision, temporary loss of local vision; some people have sensory manifestations, such as tingling in one arm or numbness^2^. Clinically, the treatment of chronic migraine subjects (CM) depends on drugs. If the symptom is relieved, preventive treatment will be used. Unfortunately, many CM patients, who frequently need to take medications, increase the frequency and severity of their headaches. This type of patient was then particularly categorized as medication-overuse headache subjects (MOH) and needs to adjust the use of medications to relieve symptoms.

Migraine can be subdivided into many categories based on the different symptoms and etiologies according to the International Classification of Headache Disorder, 3rd edition (ICHD-3). For the diagnosis of MOH, physicians can only adjust the medications based on the patient’s subjective description of their migraine attacks. Although many CM patients had overused painkillers which might worsen their migraine, they might not realize. As a result, the development of other techniques as biomarkers to classify different types of migraine is of great importance in clinical practice. This study aims to identify the relationship between near-infrared spectroscopy and migraine groups, assisting doctors in making a more accurate diagnosis.

## Results

### fNIRS signals

**Figure 1:**
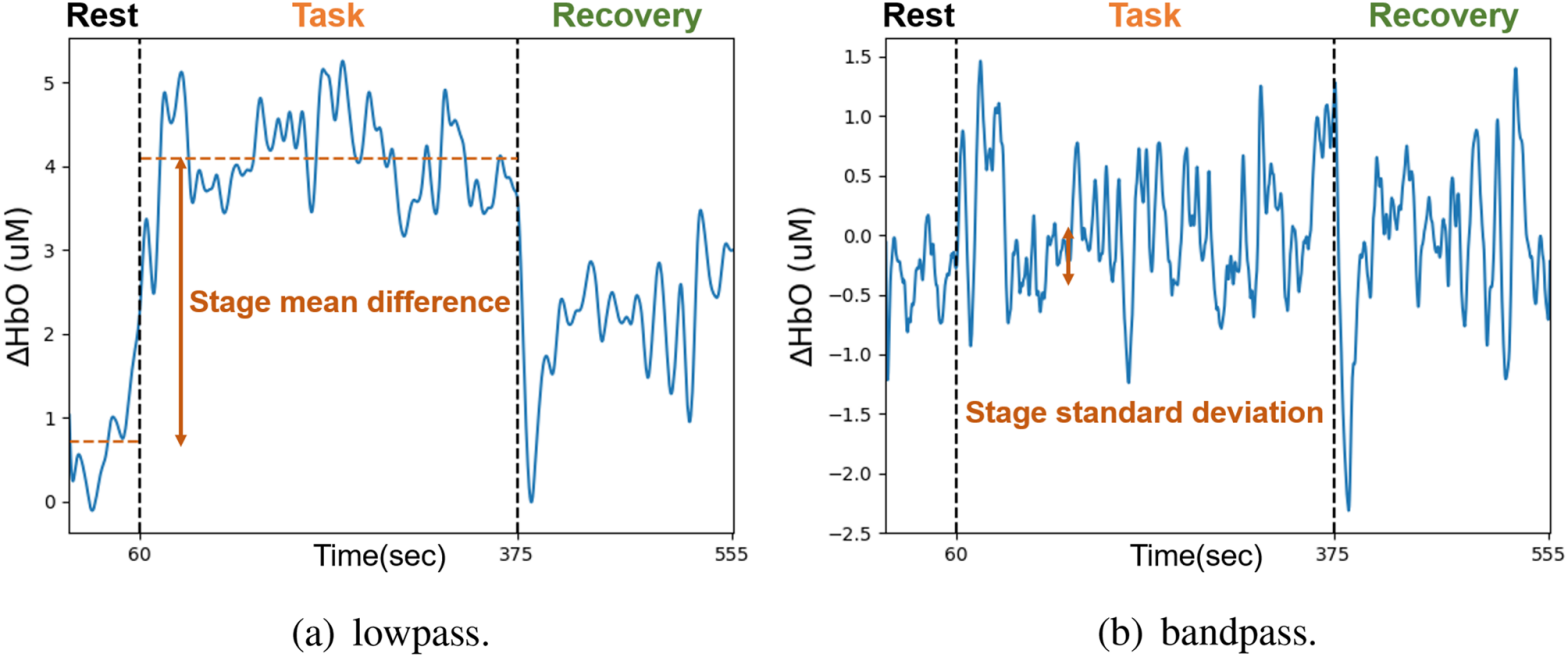
fNIRS signals during the MAT after (**a**) lowpass (**b**) bandpass filtering. This results are from one of the migraine subjects.

Figure 1 shows functional near-infrared spectroscopy (fNIRS) typical signals of one of the migraine subjects during the mental arithmetic task (MAT) . Figure 1a shows lowpass filter results and Fig. 1b shows bandpass filter results. We can observe that there is a significant change in blood oxygen concentration in the task section over time.

### LDA vs. QDA

The overall performance of QDA was better than LDA, which might be related to the hypothesis of the two algorithms. LDA assumed that the covariance matrix of each group in the model was equal, that is, the distribution of the population of each group in the feature space was the same. On the contrary, the hypothesis of QDA was looser, the covariance matrix of the different groups did not have to be the same. In this experiment, the actual feature distribution of each group might be closer to the hypothesis of QDA, which resulted in a better performance.

### Direct classification vs. stepwise classification

The classification could be divided into direct classification and stepwise classification. The former was to complete the classification of data at one time; the latter was to first divide all data into HC and Migraine and then separate CM and MOH from Migraine, which could also achieve the effect of three groups. In order to make the classification model play the greatest auxiliary benefit in the clinic, the following results chose the model with relatively high accuracy and better performance to present and discuss.

#### Direct classification

**Figure 2:**
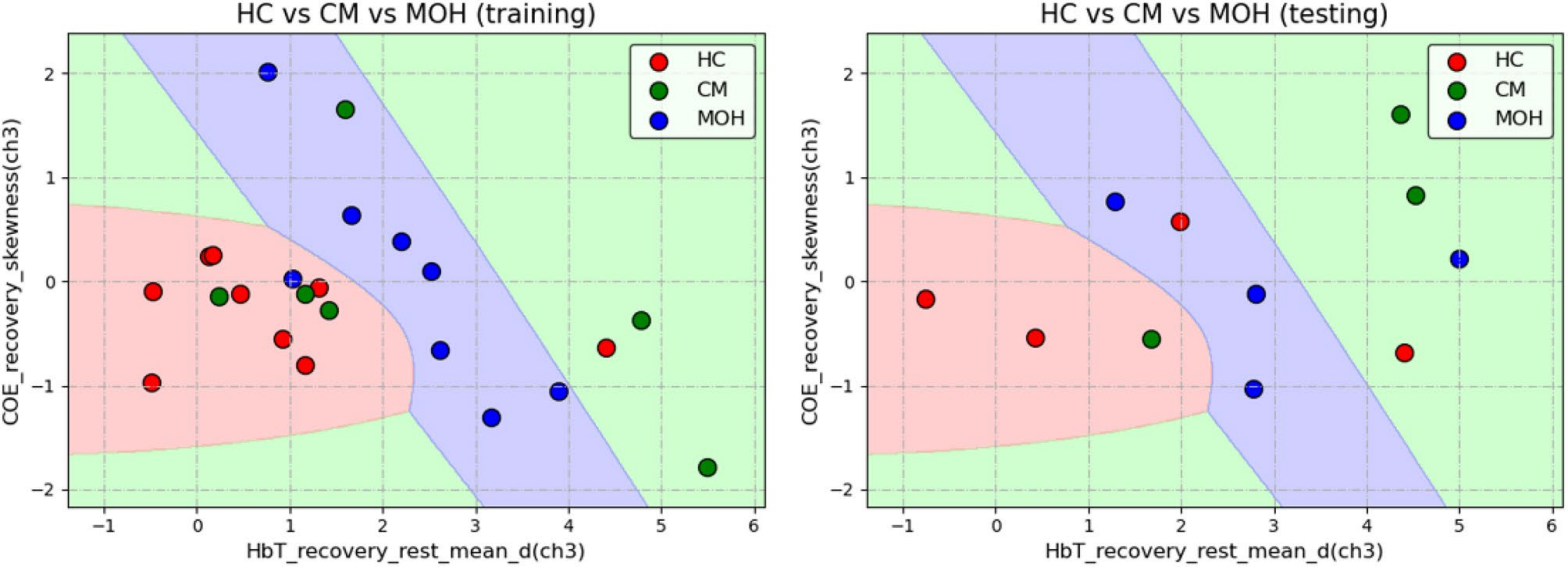
Result of direct classification.

Before training, all 34 pieces of data were divided into 23 pieces of training data and 11 pieces of test data. For the results of direct classification, the QDA algorithm was used, selecting the mean difference between the HbT of the right PFC during the recovery and rest stage (HbT_recovery_rest_mean_d (ch3)) and the skewness of the COE of the right PFC during the recovery stage (COE_recovery_skewness(ch3)) as features obtained the best results.The direct classification outcome yielded 73.9% training accuracy, 63.6% testing accuracy and 60.9% validation accuracy, which training as well as testing results shows in Fig. 2 and validation accuracy is calculated by leave-one-out cross-validation (LOOCV).

However, the 73.9% training accuracy yielded was mainly caused by the correct classification of HC and MOH, and the outcome of the model could not classify so well for CM subjects. CM subjects performed horribly . The direct classification model ideally hoped to find a set of distinguishing features among the three groups, rather than focusing on only two groups.

#### Stepwise classification

The results of the stepwise classification experiment were divided into two parts, the classification of HC and Migraine and the classification of CM and MOH. Before training the classification model of HC and Migraine, all 34 pieces of data were divided into 23 pieces of training data and 11 pieces of test data. On the other hand, before the training of the CM and MOH classification model, 21 pieces of migraine data were divided into 14 pieces of training data and 7 pieces of test data.

**Figure 3:**
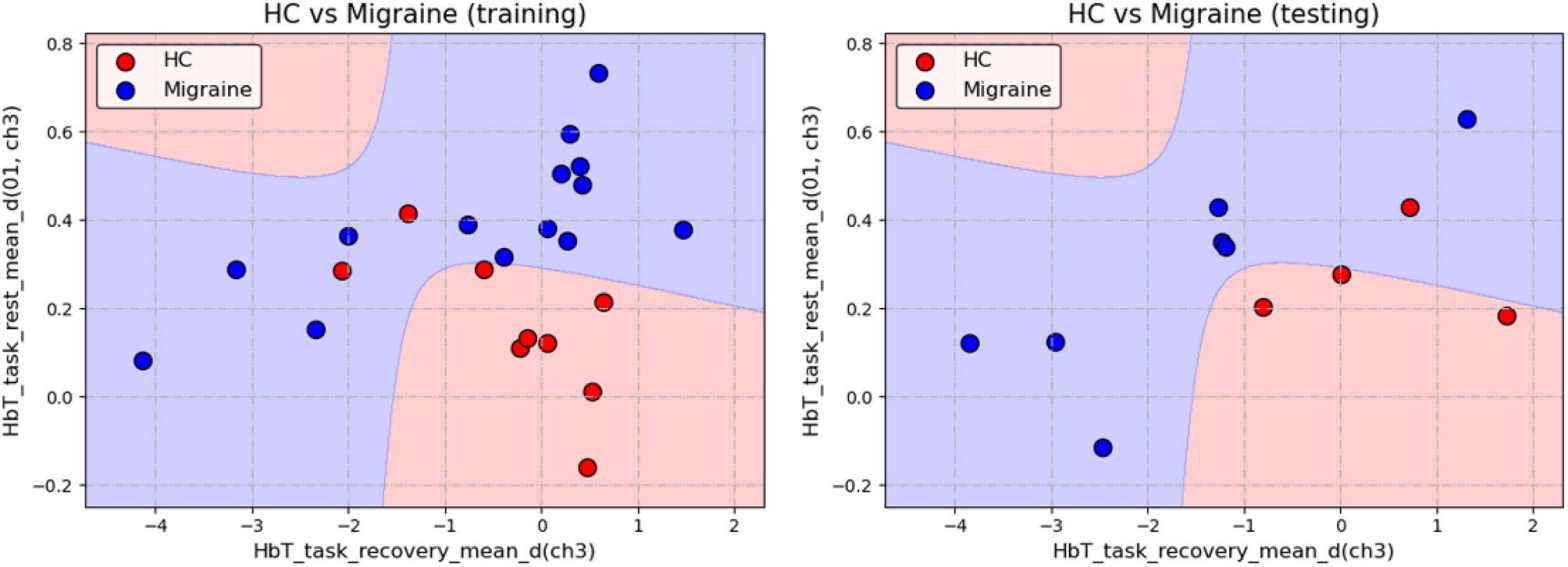
Results of Stepwise classification—classification of HC and Migraine.

**Figure 4:**
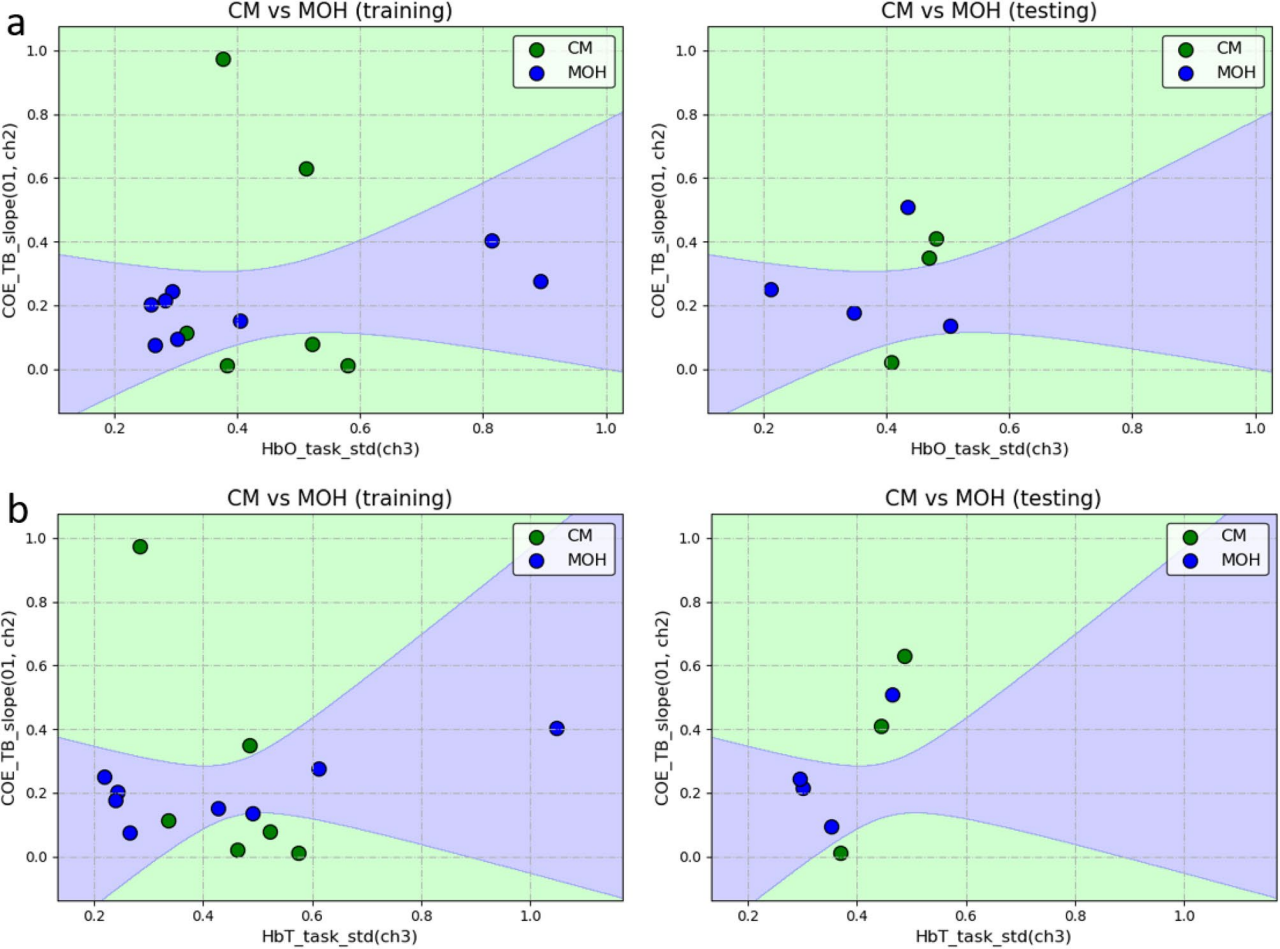
Results of Stepwise classification—classification of CM and MOH.

1. Classification of HC and Migraine In the classification results of HC and Migraine, the QDA algorithm was used, selecting the mean difference between the HbT of the right PFC in the task and recovery stage (HbT_task_recovery_mean_d (ch3)) and the trend of change of the HbT of the right PFC in the task and rest stage (HbT_task_rest_mean_d (01, ch3)) as features can get the best results (91.3% training accuracy, 87% validation accuracy, and 90.9% testing accuracy, which shows in Fig. 3). On average, the values of the two features of HC were roughly distributed between 0 to 0.1, and the value of x is near 0, which meant that for most HC, the total blood volume of the right PFC hardly changes during the task and recovery stage; The value of y is around 0.1, which meant that the total blood volume of the right PFC of most HC has increased by 10% of its own signal amplitude from the rest to the task stage; and the two features of Migraine were deviated from (0, 0.1), and the two characteristics were roughly positively correlated. When the x value was fixed, the y value of Migraine was mostly higher than HC, which meant that for Migraine, the total blood volume of the right PFC increased more than HC during the task stage. In other words, compared with HC, migraine subjects were more prone to have dilatation of local cerebral vessels after receiving task stimulation.
2. Classification of CM and MOH In the classification of CM and MOH, two sets of features had a good performance in distinguishing between CM and MOH. The first set used QDA as the algorithm, selecting the degree of dispersion of the HbO changes in the right PFC during the task stage (HbO_task_std (ch3)) and the percentage of COE changes in the left PFC at the beginning of the task (COE_TB_slope(01, ch2)) as features obtained the best result (92.9% training accuracy rate, the validation accuracy rate and the testing accuracy rate are both 85.7%, which shows in Fig. 4a). The two features of MOH were roughly positively correlated, that is, the greater the percentage change in the COE of the left PFC of MOH at the beginning of the task, the greater the fluctuation of the HbO of the right PFC during the task stage. The second set also used QDA as the algorithm, selecting the degree of dispersion of the HbT change in the right PFC during the task stage (HbT_task_std (ch3)) and the percentage change in the COE of the left PFC at the beginning of the task (COE_TB_slope (01, ch2)) as features, which quite similar to the features of the first set, also obtained a good result (The accuracy of training, validation, and testing are all 85.7%, which shows in Fig. 4b). The two features of MOH are roughly positively correlated. In addition, MOH data points are mainly distributed in the band-shaped area, while CM does not have this phenomenon.

## Discussion

**Figure 5:**
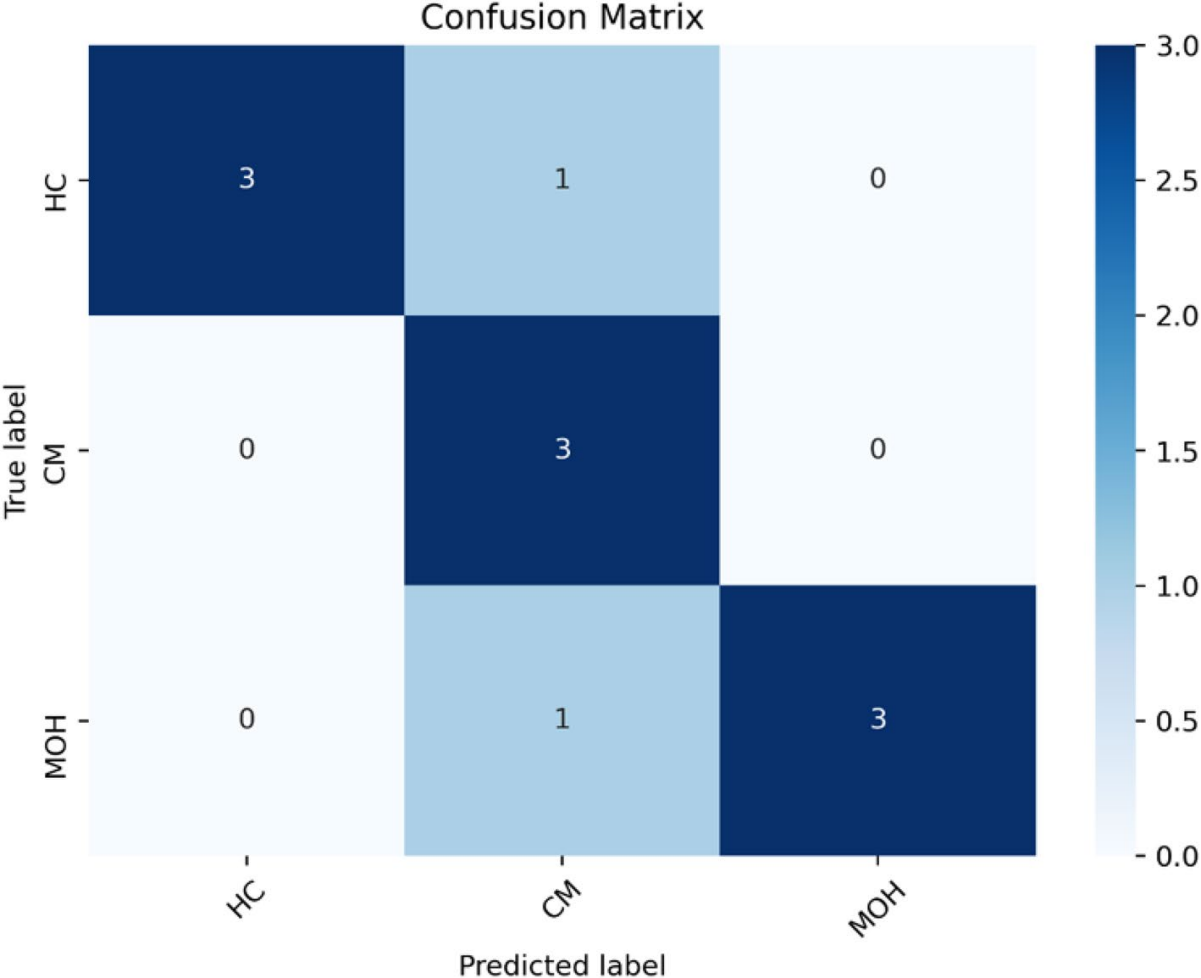
The confusion matrix of Stepwise classification—three groups.

The change of hemodynamic signals of healthy subjects was smaller while there was a large difference between each migraine patient. Therefore, in the first model, we implemented the classification of normal persons and migraineurs. Afterwards, we implemented the classification of CM and MOH in the second model. In the classification of HC and Migraine, the mean difference between the HbT of the right PFC in the task and recovery stages (HbT_task_recovery_mean_d (ch3)) and the trend of change of the HbT of the right PFC in the task and rest stages (HbT_task_rest_mean_d (01, ch3)) were features that could get the best performance. When most migraine subjects (CM + MOH) received psychological stress stimulation, the total blood volume of the right PFC increased more than that of HC, which meant that MAT produced a larger mental load for migraine subjects, and their right PFC needed more blood to cope with the oxygen consumption caused by neurometabolic^3^. It also showed that the pressure threshold of migraine subjects was lower than HC, so they were more sensitive to pressure. On the other hand, using the degree of dispersion of the HbO changes in the right PFC during the task stage (HbT_task_std (ch3)) and the percentage of COE changes in the left PFC at the beginning of the task (COE_TB_slope (01, ch2)) to classify CM and MOH had the best performance. With stepwise classification, our research indicates that it is feasible to use the NIRS signal to classify different types of migraine and control group, and the overall confusion matrix is shown in Fig. 5.

Regarding the classification of CM and MOH, the data points of MOH were distributed in the band-shaped area, while the data points of CM were scattered on both sides. The reason might be related to the formation of CM and MOH. The causes of CM are quite diverse, such as genetics, excessive caffeine intake, and insomnia^4^. These different reasons might cause CM subjects to perform differently in the same feature. On the contrary, the cause of MOH is medication overuse, and the clinical effect of medication overuse on brain regions is quite significant. Therefore, it is easy to observe that the data points were located in the band-shaped area.

However, there are still some difficulties that need to be broken through. First of all, there has little research on the application of NIRS to either MOH or CM, so the results of this experiment cannot be verified by other methods. Second, the average age of MOH analyzed in this experiment is about ten years older than that of CM subjects. Clinically, patients must first be diagnosed with a certain type of migraine, and then be diagnosed as MOH due to the change of migraine pattern caused by medication overuse. Therefore, most MOH are older. Although the selected features are not significantly correlated with age after standardization (COE_TB_slope (01, ch2): − 0.23, HbT_task_std (ch3): − 0.07, HbO_task_std (ch3): − 0.12), which can make sure the observed results are not simply an effect of age related optical or physiological changes, this problem still needs attention in the future. Last but not least, the amount of data in each group in this experiment is slightly insufficient. Since this study is a preliminary study, more subjects will be recruited in the future to overcome this problem.

## Methods

**Figure 6:**
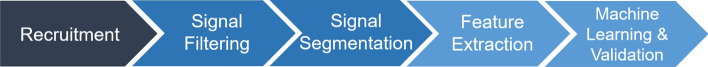
The experimental process of this study. After the fNIRS signal is obtained, it will be filtered, segmented and extracted and imported to machine learning to get classification results, and finally confirm the credibility of the results with cross-validation.

The experimental process can be divided into five steps, as shown in Fig. 6. First, we need to recruit subjects to take a Mental Arithmetic Task (MAT). When doing the task, the blood oxygen information could be measured by the device. Secondly, in order to obtain more valuable signal, signal filtering is required. Afterwards, do the signal segmentation according to the three stages of the task and do the feature extraction. Eventually, the features can be imported into the machine learning. The details will be left to the following subsections for more explanations.

### NIRS

Nowadays, more and more techniques have been investigated to explore the relationship between migraine and cerebrovascular reactivity or cerebral hemodynamics. Some studies have used positron emission tomography (PET) to scan the prefrontal cortex (PFC) and assess whether the suboccipital stimulator is effective^5^. Others have found that the ventromedial prefrontal cortex is more active in MOH than CM subjects through functional magnetic resonance imaging (fMRI)^6^. Both PET and fMRI are non-invasive imaging modalities but the former requires the application of radioactive imaging agents which lead to the concern for ionizing radiation. Although the latter does not involve radioactive agents, the use of a strong magnetic field excludes patients with an artificial pacemaker or any metal implants.

As early as 2007, there was a study using near-infrared spectroscopy (NIRS) to evaluate the difference in regional cerebral blood flow (rCBF) changes of the middle cerebral artery between migraine patients and the healthy control group during the breath-holding task^7^. In recent years, NIRS has gradually emerged in the pain field^8–11^. Moreover, NIRS has the advantages of non-invasive, non-radioactive, instant, low system cost, portability and easy operability, etc. Therefore, NIRS has an extremely high potential as a tool for investigating migraine.

### Headgear setup^12^

**Figure 7:**
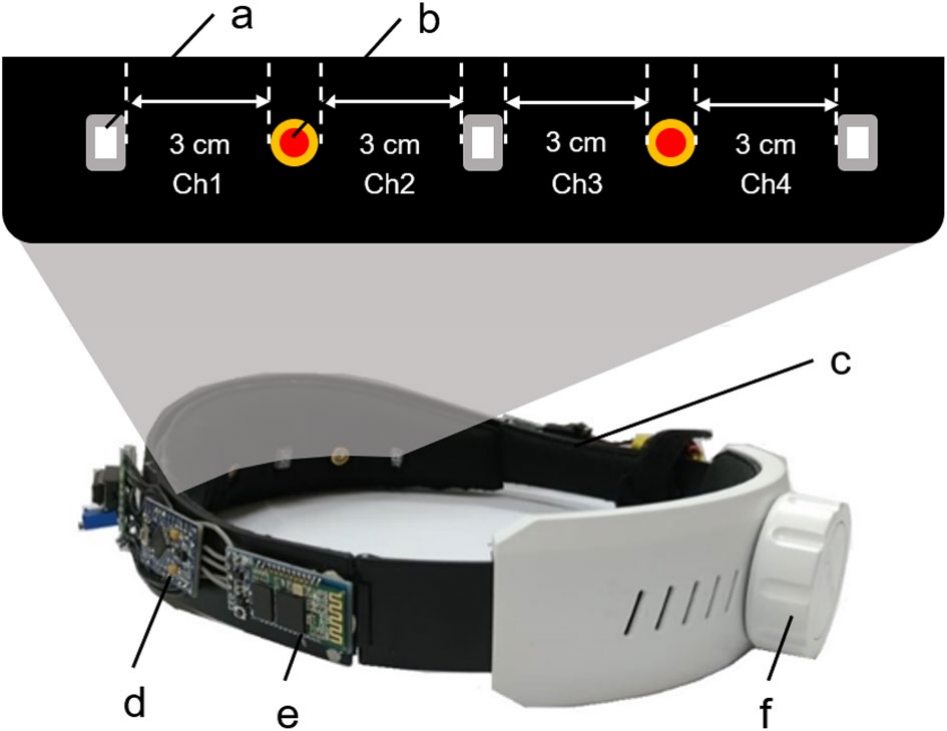
The wearable functional near-infrared spectroscopy system. (**a**) OPT101 (**b**) LED (**c**) Power source (**d**) MCU (**e**) Bluetooth module (**f**) Regulator knob.

**Figure 8:**
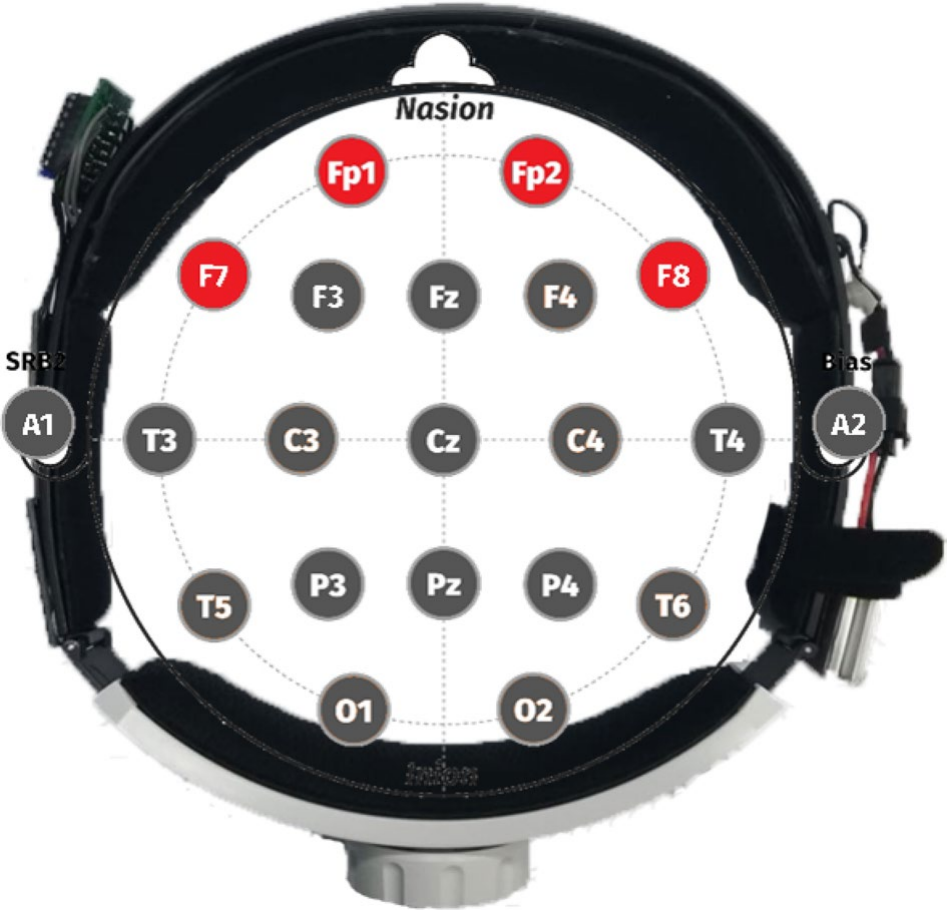
The Schematic positions of fNIRS optodes in the international 10–20 system.

The continuous-wave NIRS system used in this experiment is a self-developed instrument in our laboratory, as shown in Fig. 7. Optode is the core of the system, consisting of three light detectors and two near-infrared light emitters staggered with a spacing of 3 cm. The four channels of the system cover the PFC, approximately at the positions of F7, Fp1, Fp2, and F8 in the International 10–20 system, shown in Fig. 8. The photodetector uses OPT101 (Texas Instruments Inc), which has the advantages of small size and high sensitivity to the near-infrared light band. The multi-wavelength LEDs (Epitex Inc, L4*730/4*805/4*850-40Q96-I) contain three wavelengths of 730 nm, 805 nm, and 850 nm. In this study, we use only 730 nm and 850 nm. The sampling frequency is about 17 Hz. The rear end of the device is equipped with an adjustment knob, which can make the device fit properly, and reduce the influence of external light. The power supply of the hardware uses a rechargeable 7.4 V battery, composed of two 3.7 V lithium batteries in series, and is directly connected to the microcontroller unit (MCU), an Arduino Pro Mini. The other components (including light detectors, a Bluetooth module, and a current regulator) are powered by the output pin of the MCU. The current regulator uses TLC5916 (Texas Instruments Inc), which can provide a constant current for the LEDs in the circuit. The MCU converts the original light intensity signal into the hemoglobin value and sends these data back to the computer through Bluetooth for storage. Finally, the computer displays the hemoglobin value in real time.

### Mental arithmetic task (MAT)

**Figure 9:**
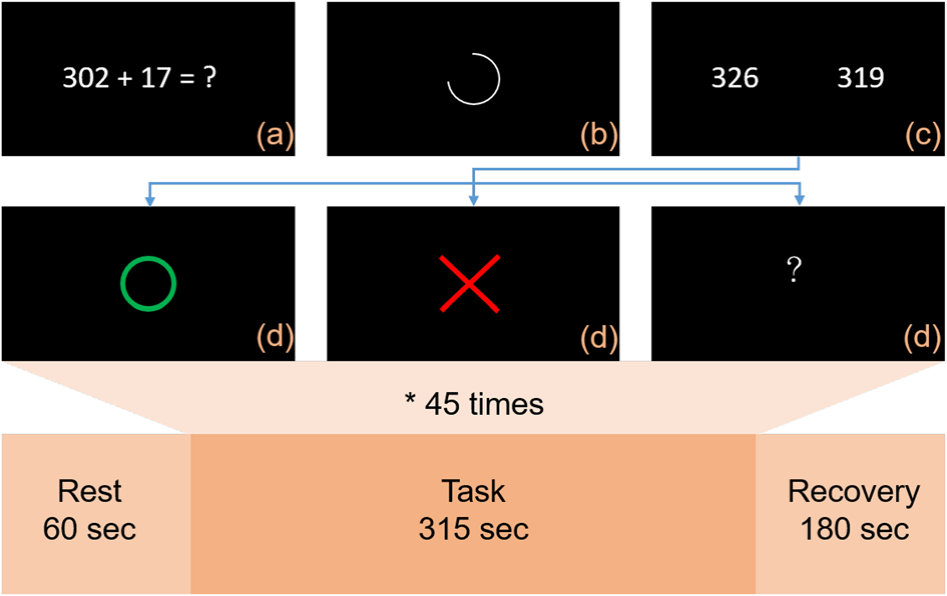
The MAT architecture. (**a**) A two-/three-digit addition/subtraction question will be displayed at the center of the screen for 1 second. (**b**) A countdown circle will be displayed on the screen for 4 seconds to remind the subject the remaining time to think. (**c**) The screen will be divided into two areas to display an answer separately. Subjects had 1 second to select the correct answer. (**d**) The screen shows a feedback for the result for 1 second. If the answer was correct, a green circle would be displayed; if the answer was wrong, a red cross would be displayed; if the correct answer was not selected in time, a white question mark would be displayed. Performing (**a**−**d**) once is a cycle, and the task stage includes 45 cycles.

MAT is a common and effective stress task. Research has confirmed that the MAT can produce mental stress in healthy subjects^13,14^ or migraine subjects^15^. Subjects were arranged in a quiet space to avoid interference from the outside world, informed of the process, and given a short practice opportunity to eliminate the experimental deviation due to unfamiliarity with operation. The MAT architecture was divided into three stages (Rest, Task, and Recovery) with a total duration of 555 s^16^, which shows in Fig. 9. At the rest stage, subjects were asked to close their eyes and relax in the seat for 1 minute. At the task stage, subjects were asked to watch the questions and answer through a touch screen. At the recovery stage, subjects had to do the same things as the rest stage for 3 minutes. The computer saved the data in the form of comma-separated values after the completion of the MAT.

### Recruitment

Recruitment was started only after the approval of the Institutional Review Board (IRB) of the Taipei Veterans General Hospital (No.: 2017-01-010C). All methods in this research were performed in accordance with the relevant guidelines and regulations. The inclusion criteria are subjects from 20 to 60 years old, meeting the diagnostic criteria of the third edition of the International Headache Classification (ICHD-3), and those can fully record the migraine attack pattern and basic personal data. Exclusion criteria are those with any major mental or neurological diseases (including brain damage, brain tumors), smoking habits or alcohol abuse. HC include 13 medical staff of Taipei Veterans General Hospital with an average age of 44.9 ± 8.7 years old. Both CM and MOH are patients in the Neurology Clinic of Taipei Veterans General Hospital. There are 9 and 12 patients with an average age of 34.8 ± 10.9 years old and 45.8 ± 11.2 years old respectively. Informed consent was obtained from all subjects.

### Signal filtering

The signal of fNIRS can be divided into three aspects: (i) source (intracerebral vs. extracerebral), (ii) stimulus/task relation (evoked vs. non-evoked), and (iii) cause (neuronal vs. systemic)^17^. In our study, task-evoked neurovascular coupling and spontaneous neurovascular coupling is of primary interest. In order to obtain different types of fNIRS signals for subsequent feature extraction, two different filters were used in parallel in this procedure. The first was the Low-pass filter, a fourth-order Butterworth filter, with a cutoff frequency of 0.1 Hz^18^, which could filter out systemic noise such as breathing, heartbeat, and Mayer wave, which was 1 Hz, 0.3 Hz, 0.1 Hz respectively. Then the changes of neurovascular coupling signal caused by the entire MAT can be obtained. The second was a band-pass filter with a frequency band of 0.01 Hz−0.3 Hz^19^. The hemodynamics response of the PFC, the signal changes after every stimulation, could be observed.

### Feature extraction^20,21^

As the purpose of MAT was to stimulate the PFC, the corresponding two channels, Ch2 and Ch3, were focused on. The collected signals included oxygenated hemoglobin (HbO) and deoxygenated hemoglobin (HHb). In addition, two different signals could be obtained by adding or subtracting these two signals, total hemoglobin (HbT) and brain oxygen exchange (COE) respectively. These data were divided into three parts by different stages of MAT (rest, task, recovery).

**Figure 10:**
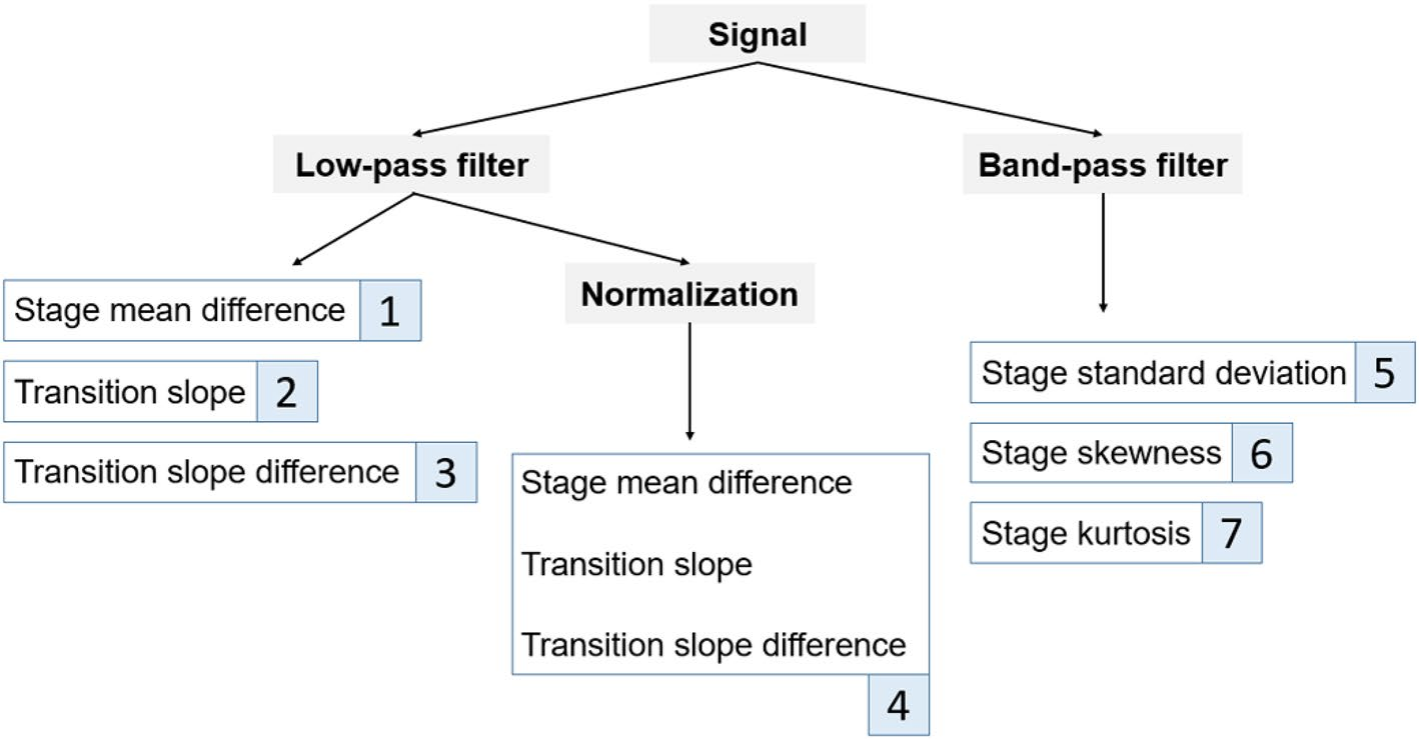
Diagram of features.

Feature extraction is a method of sorting out available features from a large range of data. Proper feature extraction will improve the quality of model training. The features used in the experiment, demonstrating in Fig. 10, will be introduced one by one below

- Low-pass filter
  1. Stage mean difference The average difference of hemoglobin at each stage. In order to observe the average change of fNIRS signal of the subject at different stages.
  2. Transition slope Referring to the article published by Coyle et al.^22^ in 2004, which is mentioned that the maximum value of light intensity can be detected by fNIRS at about five to eight seconds after stimulation, so we took the maximum value of eight seconds. The slope of the fNIRS signal when the first eight seconds after entering a new stage . Fitting the value of the interval with a linear formula, and the coefficient of the first term is the slope. In order to observe the changes of the fNIRS signal under different stimulation.
  3. Transition slope difference The difference of transition slope. In order to observe the difference in the changes of the fNIRS signal under different stimulation.
  4. Normalization Normalization is a procedure for moving and rescaling data. Feature 1 $\sim$ 3 were calculated again after this process. The normalized data fall between zero and one, which could compare the differences in the ratio of the characteristics of fNIRS signal among the subjects to the changes in their own signal amplitude.
- Band-pass filter
  1. Stage standard deviation The standard deviation of the fNIRS signal at each stage. In order to observe the dispersion level of data.
  2. Stage skewness The skewness of fNIRS signal at each stage. In order to observe the asymmetry of the distribution of the signal value.
  3. Stage kurtosis The kurtosis of the fNIRS signal at each stage, which described the tail length of the distribution of the signal value^23^. Compared with the value near the average, outliers had a greater impact on the value of kurtosis.

Combining the above-mentioned features, a total of 144 features were obtained. These features were the inputs of linear discriminant analysis (LDA) and quadratic discriminant analysis (QDA).

### Machine learning

Logistic regression is a model commonly used for classification, but it has some disadvantages. First, logistic regression can only deal with the problem of two classifications, and it will be tricky when it encounters multiple classifications; second, it cannot handle well when faced with a large number of features or variables. The most important thing is that if the amount of data is too small, the results will be unstable due to a lack of basis for optimizing parameters. LDA can offset this disadvantage, especially multi-group performance. LDA has two basic hypotheses. First, the algorithm assumes that each group of data is Gaussian distribution. Second, in order to make the decision boundary have a clear geometric meaning, the covariance matrix of each group of data must be equal. On the other hand, QDA does not have the limitation of covariance matrix. In addition, the credibility of the model was evaluated by leave-one-out cross-validation (LOOCV), which was often used in the small data set and made the performance of the fNIRS diagnostic ability more confident.
