# Author Correction: Migraine classification by machine learning with functional near-infrared spectroscopy during the mental arithmetic task

**DOI:** 10.1038/s41598-024-56279-9

**Published:** 2024-03-13

**Authors:** Wei‑Ta Chen, Cing‑Yan Hsieh, Yao‑Hong Liu, Pou‑Leng Cheong, Yi‑Min Wang, Chia‑Wei Sun

**Affiliations:** 1 Department of Neurology, Keelung Hospital, Ministry of Health and Welfare, No. 268, Xin 2nd Rd., Xinyi Dist, Keelung, 20148 Taiwan, ROC; 2 Neurological Institute, Taipei Veterans General Hospital, No. 201, Sec.2, Shipai Rd., Beitou Dist, Taipei, 112201 Taiwan, ROC; 3 Department of Photonics, College of Electrical and Computer Engineering, National Yang Ming Chiao Tung University, No. 1001, University Road, East District, Hsinchu, 300093 Taiwan, ROC; 4 Department of Pediatrics, National Taiwan University Hospital, Hisnchu Branch, No. 25, Ln. 442, Sec.1, Jingguo Rd., North Dist, Hsinchu, 30059 Taiwan, ROC; 5 Institute of Biomedical Engineering, College of Electrical and Computer Engineering, National Yang Ming Chiao Tung University, No. 1001, University Road, East District, Hsinchu, 300093 Taiwan, ROC; 6 Medical Device Innovation and Translation Center, National Yang Ming Chiao Tung University, No. 155, Sec. 2, Linong St., Beitou Dist., Taipei, 112304 Taiwan, ROC; 7 Department of Biological Science and Technology, National Yang Ming Chiao Tung University, Hsinchu, Taiwan

Correction to: *Scientific Reports* 10.1038/s41598-022-17619-9, published online 26 August 2022

The original version of this Article omitted an affiliation for Pou-Leng Cheong. The correct affiliations are listed below.

Department of Photonics, College of Electrical and Computer Engineering, National Yang Ming Chiao Tung University, No. 1001, University Road, East District, Hsinchu, 300093, Taiwan, ROC

Department of Pediatrics, National Taiwan University Hospital, Hisnchu Branch, No. 25, Ln. 442, Sec.1, Jingguo Rd., North Dist, Hsinchu, 30059, Taiwan, ROC

Department of Biological Science and Technology, National Yang Ming Chiao Tung University, Hsinchu, Taiwan

The original Article has been corrected.

